# Preparation of a Sepia Melanin and Poly(ethylene-*alt*-maleic Anhydride) Hybrid Material as an Adsorbent for Water Purification

**DOI:** 10.3390/nano8020054

**Published:** 2018-01-23

**Authors:** Guido Panzarasa, Alina Osypova, Giovanni Consolati, Fiorenza Quasso, Guido Soliveri, Javier Ribera, Francis W. M. R. Schwarze

**Affiliations:** 1 Department of Polymer Engineering and Science, Montanuniversität, Otto-Glöckel Straβe 2, 8700 Leoben, Austria; 2 Laboratoire de Réactivité de Surface, Sorbonne Universités, UPMC Univ Paris 06, F-75005 Paris, France; alina.osypova@gmail.com; 3 Department of Aerospace Science and Technology, Politecnico di Milano, via La Masa 34, 20156 Milano, Italy; giovanni.consolati@polimi.it (G.C.); fiorenza.quasso@polimi.it (F.Q.); 4 INFN, Sezione di Milano, via Celoria 16, 20133 Milano, Italy; 5 Department of Chemistry, Université de Montréal, C.P. 6128 Succ. Centre-ville, Montréal, QC H3C 3J7, Canada; guido.soliveri@gmail.com; 6 Empa Materials Science and Technology, Laboratory for Applied Wood Materials, Lerchenfeldstrasse 5, 9014 St. Gallen, Switzerland; javier.ribera@empa.ch (J.R.); francis.schwarze@empa.ch (F.W.M.R.S.)

**Keywords:** sepia melanin, poly(ethylene-*alt*-maleic anhydride), hybrid materials, adsorbent, gel, water purification, methylene blue

## Abstract

Meeting the increasing demand of clean water requires the development of novel efficient adsorbent materials for the removal of organic pollutants. In this context the use of natural, renewable sources is of special relevance and sepia melanin, thanks to its ability to bind a variety of organic and inorganic species, has already attracted interest for water purification. Here we describe the synthesis of a material obtained by the combination of sepia melanin and poly(ethylene-*alt*-maleic anhydride) (P(E-*alt*-MA)). Compared to sepia melanin, the resulting hybrid displays a high and fast adsorption efficiency towards methylene blue (a common industrial dye) for a wide pH range (from pH 2 to 12) and under high ionic strength conditions. It is easily recovered after use and can be reused up to three times. Given the wide availability of sepia melanin and P(E-*alt*-MA), the synthesis of our hybrid is simple and affordable, making it suitable for industrial water purification purposes.

## 1. Introduction

Life—or, at least, human life—is not possible without water. Despite this common knowledge, however, access to sources of clean, drinkable water cannot be taken for granted worldwide due to both natural and anthropogenic factors [1]. The latter one comprises the misuse and abuse of water, such as improper or no treatment of industrial effluents from highly polluting processes—e.g., mining and textile dyeing—which are extremely rich in toxic metals and organic compounds, or of black waters from human settings, which are loaded with pesticides, drug residues, and other contaminants [2]. 

To meet the increasing demand of drinkable water, several wastewater treatment technologies have been proposed at laboratory as well as field levels. Methods based on adsorption are usually preferable to those based on precipitation, being more versatile and allowing for easier management of the waste. Thus it is crucial to develop efficient and sustainable adsorbent materials [3]. It is also important to ensure that the spent adsorbents could be disposed of without the generation of additional waste. In this context, the focus is on materials and composites derived from natural and renewable resources, an approach which has its roots in biomass valorization [4]. 

Melanin(s), a heterogenous class of widespread natural pigments [5], can be considered a promising source of new materials thanks to natural abundance, bio-renewability, low cost, and specific physicochemical properties. One of the most studied melanins is the dye extracted from the ink of cuttlefish (*Sepia officinalis* L.), thus called sepia melanin. Despite decades of dedicated research, the final word still has to be said regarding its exact chemical and supramolecular constitution, hindering progress on the development of new materials based on this melanin. Nevertheless, its nature as a product of oxidative polymerization of 3,4-dihydroxyphenylalanine (DOPA) or similar precursors (such as dopamine) has been demonstrated, together with the presence of catechol, quinone, and indole moieties [6,7]. Moreover, its ability to adsorb and chelate a wide range of ions and molecular substances (including heavy metals, radioisotopes, pesticides, and drugs) is recognized, which suggests the suitability of sepia melanin for detoxification and water purification purposes [8,9]. 

Poly(ethylene-*alt*-maleic anhydride) (P(E-*alt*-MA)) has been known since the mid-1940s and proved useful as reactive compatibilizer for polymer blends [10], as dispersing aid for solids [11], and for more advanced applications including biosurface engineering [12] and gate insulators [13]. In moderate alkaline environments, each anhydride moiety transforms into two carboxylate groups, making the polymer a water-soluble polyelectrolyte with interesting chelating properties. However, its practical use for water remediation is impaired by its high solubility, making its removal (e.g., by means of membrane filtration [14]) inconvenient from a practical perspective. Polymer crosslinking—e.g., by the reaction of anhydride groups with bifunctional amines—has been described as a convenient way to achieve water insolubility while maintaining good chelating properties [15]. 

In the present study, we demonstrate how sepia melanin can be chemically combined with poly(ethylene-*alt*-maleic anhydride), giving rise to a stable, water-insoluble, and highly efficient hybrid adsorbent material. 

## 2. Results and Discussion

### 2.1. Sepia Melanin-P(E-alt-MA) Hybrid: Its Synthesis and Characterization

**Figure 1:**
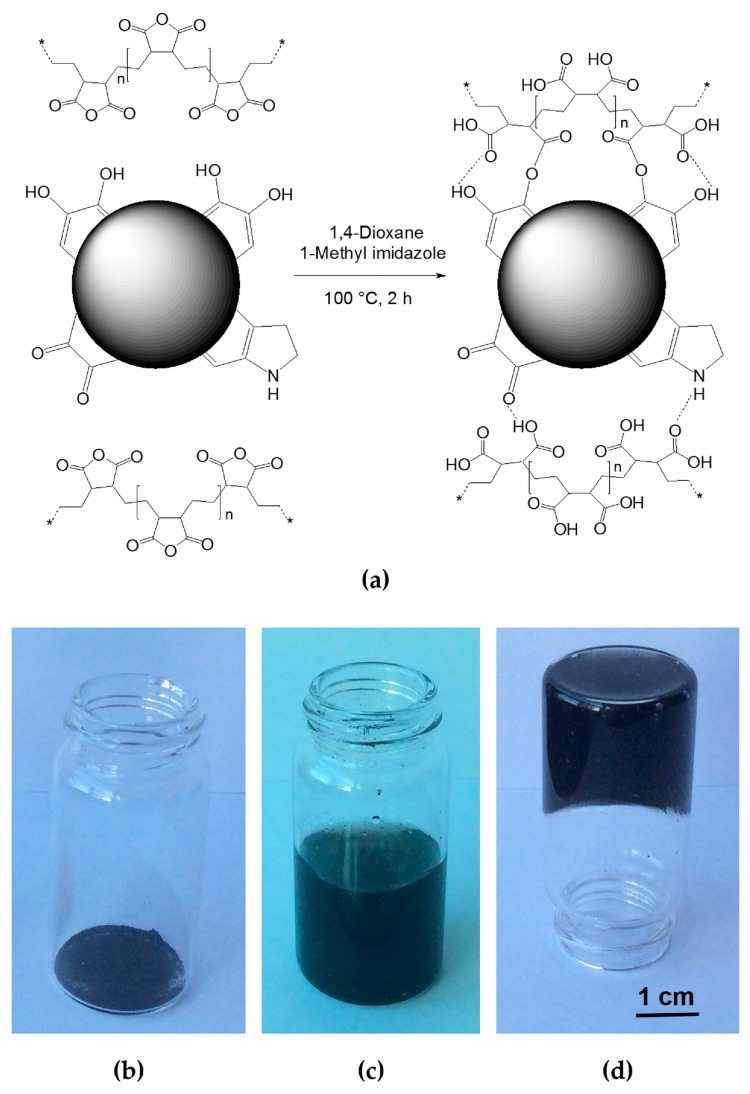
(**a**) Schematic representation of the synthetic approach and proposed structure for our sepia melanin-P(E-*alt*-MA) hybrid. Melanin is represented as a black pearl with protruding functional groups and dashed lines represent hydrogen bonds; (**b**) Photograph of the as-prepared hybrid; (**c**,**d**) Photographs of the gel obtained upon addition of water to powdered hybrid.

We speculated on the possibility of forming chemical bonds between the catechol groups of sepia melanin and the carboxylic acid moieties of poly(ethylene-*alt*-maleic anhydride). The proposed formation and structure of our hybrid is illustrated in Figure 1a. The actual formation of covalent bonds, other than just hydrogen bonds, hydrophobic, or electrostatic interactions, between sepia melanin and P(E-*alt*-MA) under the explored conditions was indirectly proved in two ways: (1) The obtained hybrid is not soluble even in concentrated alkali (5 M NaOH) while P(E-*alt*-MA) is extremely soluble; (2) The reaction was carried out in absence of sepia melanin: only a small amount of a gelatinous product was obtained upon precipitation with water, which readily dissolved upon the addition of NaOH.

1,4-Dioxane was chosen for being a good solvent of P(E-*alt*-MA) and for its miscibility with water, which facilitated the recovery of the product. 1-Methylimidazole is a well-known catalyst for esterification reactions involving anhydrides. The sepia melanin-P(E-*alt*-MA) hybrid is obtained as a black solid (Figure 1b) after extensive washing with water and dehydration with acetone. Acetone as a dehydrating agent helped to remove solvent residues and transformed an otherwise voluminous gel in an easy-to-handle powder. 

We observed that our hybrid readily absorbs water and forms a gel (Figure 1c,d). Such a behavior can be easily rationalized by comparison with similar adsorbing polymers based on, e.g., crosslinked poly(acrylic acid) [16]. For hydrogels, the gelation capability is usually expressed by the critical gelation concentration (CGC), defined as the minimum amount of gelator required to gelate 1 mL of water [17]. For the material investigated here, the CGC was determined by the vial inversion method to be around 5 mg mL^−1^. By means of lyophilization, this hydrogel could be easily converted in lightweight foam. 

**Figure 2:**
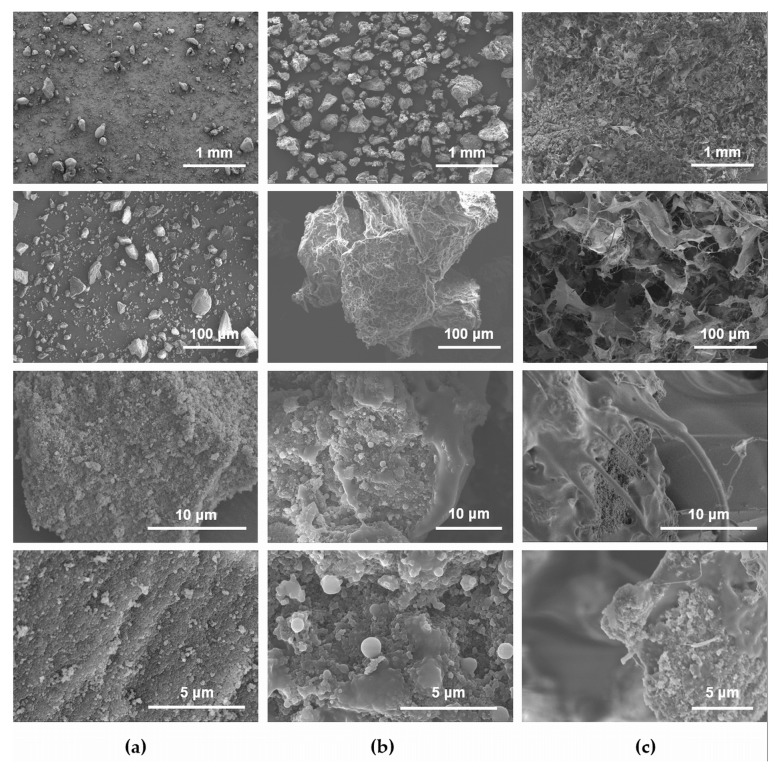
Scanning electron micrographs of (**a**) sepia melanin; (**b**) as-prepared sepia melanin-P(E-*alt*-MA) hybrid; and (**c**) hybrid’s lyophilized gel.

Further insight on the physicochemical properties of the synthesized hybrid was obtained by means of different analytical techniques. A morphological investigation of sepia melanin, of the as-prepared hybrid and of its lyophilized gel was carried out by scanning electron microscopy (SEM) and the results are shown in Figure 2. Under the microscope, the sepia melanin sample is made of polydisperse particles with irregular shapes and micrometer size (Figure 2a, Figure S1). At higher resolution, these macroparticles appear to be aggregates of smaller, spherical particles with an average diameter of ~200 nm, which is in perfect agreement with literature findings [18]. The sepia melanin-P(E-*alt*-MA) hybrid appears as well to be made of micrometer-sized particles (Figure 2b), but images taken at higher magnification show that melanin particles are embedded in what can be considered a polymer matrix. This matrix is the same that generates, in the hybrid’s lyophilized gel (Figure 2c), a network structure where melanin aggregates are still visible. 

A semiquantative elemental analysis for carbon, oxygen, and nitrogen was performed by means of energy-dispersive X-ray spectroscopy (EDX). The elemental percentages obtained, as well as the corresponding C/O and C/N ratios, are summarized in Table 1. 

For P(E-*alt*-MA), the theoretical C/O ratio is 2. On the other hand, the theoretical C/O ratio for its free acid (hydrolyzed) form is 1.5, which is closer to our experimental result. This is reasonable, since EDX probed the surface of a powder granule, which upon exposure to the open atmosphere could have rapidly hydrolyzed. In any case, due to the chosen experimental conditions, we expect the anhydride groups of the P(E-*alt*-MA) entering in the composition of our hybrid to be fully hydrolyzed. To find reference values for sepia melanin is more difficult, given its known variability in composition. Chedekel et al. [19] reported a C/O ratio of 1.7 and a C/N of 7.67 for sepia melanin, which are in good agreement with our findings, suggesting a higher proportion of sepia melanin than of P(E-*alt*-MA) entering in the composition of our hybrid.

Little information could be extracted from Fourier transform infrared (FTIR) spectroscopy (Figure S2). Neither the spectrum of sepia melanin nor that of the synthesized hybrid had features of significant diagnostic value, as it is usually observed for melanins [20]. Nevertheless, the FTIR spectra revealed more similarities between sepia melanin and the hybrid than between the latter and P(E-*alt*-MA). A slight broadening of the peak at 1738 cm^−1^ in the spectrum of the hybrid could be indicative of the presence of different carbonyl species. Small differences could be observed also in the aliphatic region (2830–3070 cm^−1^). 

**Figure 3:**
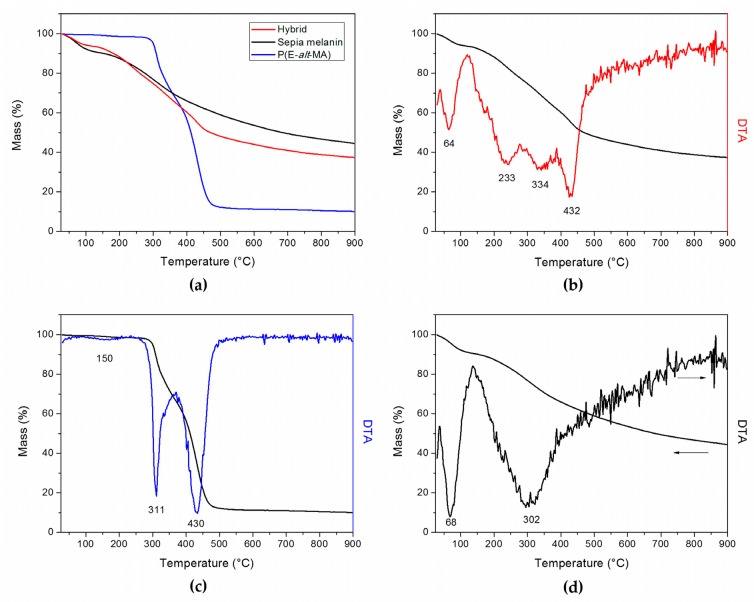
(**a**) Summary of the thermogravimetric plots; (**b**) TGA and DTA analysis of the sepia melanin-P(E-*alt*-MA) hybrid; (**c**) TGA and DTA analysis of P(E-*alt*-MA); (**d**) TGA and DTA analysis of sepia melanin.

The thermal behavior was investigated by means of differential scanning calorimetry (DSC) and thermogravimetric analysis (TGA). DSC showed a reversible endothermic transition for P(E-*alt*-MA) associated with melting, but failed to show any relevant transition in the investigated temperature interval, except for water desorption during the first cycle, for both sepia melanin and the hybrid. TGA was far more informative: the results, shown in Figure 3, suggest that the thermal behavior of our hybrid somewhat combines those of the precursors, resembling more closely sepia melanin in the broad decomposition range extending from 150 to 500 °C. It is clear from the first derivative of the TGA curve (DTA) that our hybrid suffers at least four different mass losses (Figure 3b). The first loss at 64 °C and the last at 432 °C can be assigned respectively to the release of weakly-bonded water and polymer decomposition. The losses at 233 °C and 334 °C may be consistent with the release of strongly-bound water and the decomposition of sepia melanin [21]. From the residual mass values (Table 2), it can be estimated that the content of P(E-*alt*-MA) in the hybrid is approximately 20%. 

This is in good agreement with the SEM, EDX, and FTIR results, confirming that the hybrid consists mainly of sepia melanin. All these observations, together with the calculated product yield and the fact that no losses of melanin were observed during the preparation of the hybrid (the supernatant of the reaction mixture being transparent and colorless), can be reasonably explained by assuming that only a relatively small fraction of the P(E-*alt*-MA) employed for the reaction entered in the composition of the hybrid.

### 2.2. Evaluation of the Adsorption Efficiency

**Figure 4:**
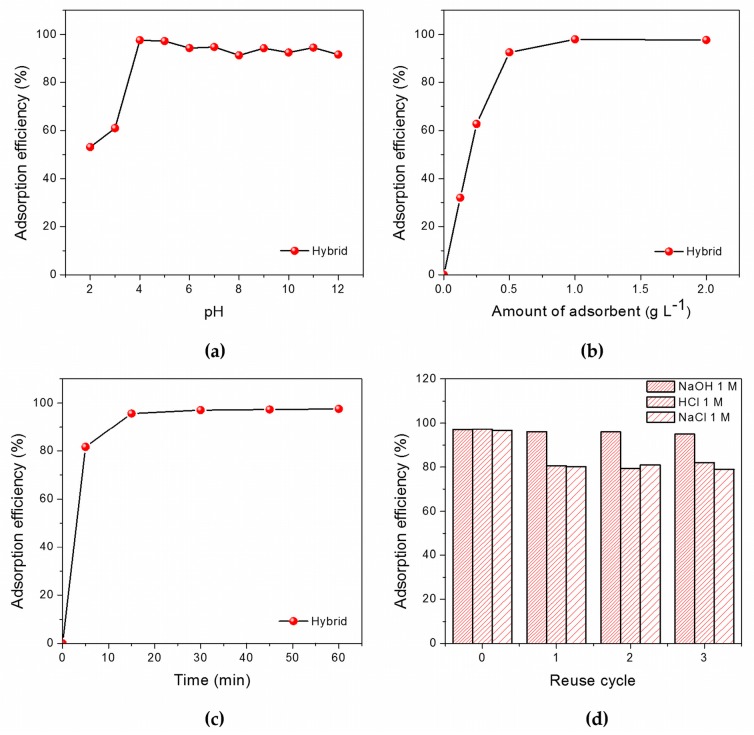
Evaluation of the adsorption efficiency of the sepia melanin-P(E-*alt*-MA) hybrid (**a**) as a function of pH; (**b**) as a function of the adsorbent’s amount; (**c**) as a function of time; (**d**) as a function of reuse cycle with different desorbing agents. All data shown are the average of three independent measurements, the relative standard deviation being ≤10%. The lines are a guide for the eye.

The adsorption efficiency of our sepia melanin-P(E-*alt*-MA) hybrid was evaluated against methylene blue (MB). This organic dye was chosen as a model organic pollutant thanks to its favorable properties: solubility in water, good chemical stability over a wide range of pH, and wide industrial use. The affinity of DOPA-melanins for methylene blue and, in general, for phenazine dyes, is well-known [22]: they are routinely used for staining melanosomes and for the photodynamic therapy of melanoma [23]. The affinity of melanin for MB is explained in terms of electrostatic interaction and π-π stacking [22]. As is shown in Figure S3, the maximum efficiency of adsorption of sepia melanin for MB is at pH 7. On the other hand, our hybrid is active in a wider pH range, with a peak of activity (adsorption efficiency 98%) at pH 4 (Figure 4a). This is in good agreement with the pK_a_ of hydrolyzed P(E-*alt*-MA), which, according to the measurements of Bianchi et al. [24], is around 3.65. In correspondence of the pK_a_ value, the carboxylic acid groups in hydrolyzed P(E-*alt*-MA) are deprotonated and negatively charged, maximizing the interaction with the positively charged MB. 

As shown in Figure 4b, a 1 g L^−1^ concentration of hybrid is enough to ensure the complete MB absorption. Moreover, our hybrid is a rapid adsorbent, reaching the peak efficiency after only 15 min at pH 4 (Figure 4c). Another important parameter for adsorbent materials is the possibility of reusing them after regeneration by controlled desorption. For our hybrid, the effect of both pH and ionic strength was tested. The results, summarized in Figure 4d, demonstrate that our hybrid maintains high adsorption efficiency, even after three cycles of adsorption–desorption. The latter can be achieved in a short time (10 min) using concentrated solutions of NaOH, HCl or even NaCl. 

## 3. Materials and Methods 

Poly(ethylene-*alt*-maleic anhydride) (avg. M_w_ 100,000–500,000 g mol^−1^), 1-methylimidazole (≥99%, purified by redistillation), 1,4-dioxane (≥99%, ACS reagent), acetone (HPLC grade), and methylene blue were purchased from Sigma-Aldrich (Buchs, Switzerland) and used as received. Water obtained from a MilliQ purification system (Merck Millipore, Schaffhausen, Switzerland) was thoroughly used. A phosphate buffer (PB) solution was prepared by dissolving 1.42 g Na_2_HPO_4_ and 0.24 g KH_2_PO_4_ in 1 L of water. Sepia melanin was extracted from commercial cuttlefish (*Sepia officinalis* L.) ink according to Zhang et al. [25]. Briefly, the ink was centrifuged at 6000 rpm for 15 min to remove aggregates and debris. The supernatant was first adjusted to pH 13 with 10 M NaOH to solubilize the pigment, then to pH 2 with 5 M HCl. The precipitated melanin was centrifuged at 12,000 rpm for 30 min to remove the supernatant and then the precipitate was dissolved in 10 M NaOH. Chloroform was used to roughly deproteinize the extract. After centrifugation, the pigment was precipitated at pH 2 and collected by centrifugation. The obtained melanin was washed first with methanol, then with 70% ethanol followed by water and eventually air-dried.

Attenuated total reflectance Fourier transform infrared spectroscopy (ATR-FTIR) was performed using a Nicolet™ iN™10 (Thermo Fisher Scientific, Reinach, Switzerland) equipped with a liquid nitrogen-cooled MCT detector (Thermo Fisher Scientific, Reinach, Switzerland) (spectral range 4000–700 cm^−1^, resolution 4 cm^−1^); background (500 scans) was collected before each sample (1000 scans). UV–vis absorption spectra were obtained using a Cary 50 Bio UV–visible spectrophotometer (Varian, Mulgrave, Australia). Scanning electron microscopy (SEM) and energy dispersive X-ray spectroscopy (EDX) were carried out using a Hitachi S-4800 scanning electron microscope (Hitachi, Tokyo, Japan) equipped with an INCA X-Sight EDS (Oxford Instruments, Abingdon, UK) detector. The samples were fixed on conductive carbon tape and sputter-coated with 5 nm of Au/Pd alloy to facilitate imaging. Thermogravimetric analysis (TGA) was performed with a Netzsch TG 209 F1 Iris instrument (Netzsch, Selb, Germany) on 5 mg-samples in alumina crucibles under a nitrogen atmosphere (flow rate 25 mL min^−1^), scanning a temperature range from 25 to 900 °C with a heating rate of 10 °C min^−1^. Differential scanning calorimetry (DSC) measurements were carried out by means of a Mettler Toledo DSC 822e instrument (Mettler-Toledo, Langacher, Switzerland) calibrated with high purity standards (indium and zinc). Samples of 3.5–5 mg were encapsulated in aluminum pans and heated from 20 to 250 °C at a rate of 10 °C min^−1^ under nitrogen flux. For each sample, two heating and cooling cycles were performed. 

### 3.1. Synthesis of the Sepia Melanin-P(E-alt-MA) Hybrid

In a 250 mL three-necked round-bottomed flask, equipped with a water condenser, 2.5 g of poly(ethylene-*alt*-maleic anhydride) were dissolved in a mixture of 30 mL of 1-methyl imidazole and 15 mL of dioxane under stirring. Then, 2.5 g of sepia melanin, previously ground to a fine powder in a mortar, were suspended in 85 mL of dioxane and added to the solution in the flask. The mixture was refluxed at 100 °C under stirring for 2 h and then allowed to cool to room temperature. The supernatant was discarded and water was added directly to break the jelly precipitate forming a loose gel which was then collected by filtration or centrifugation and washed repeatedly with water until the washings were colorless. Then, the solid was dispersed in 300 mL of water under stirring overnight to complete the washing. Eventually, the solid was dehydrated with acetone to obtain a fine powder which was dried under vacuum overnight. The yield was 2.95 g (59% on mass basis).

To obtain a foam, water (e.g., 10 mL) was added with manual mixing to a proper amount of powdered sample (e.g., 50 mg) until a stable gel was formed, which was then lyophilized. 

### 3.2. Adsorption Studies

#### 3.2.1. Effect of pH on Adsorption Efficiency

The effect of pH on the adsorption efficiency was studied using a 1 g·L^−1^ amount of adsorbent. A batch solution containing 50 mg L^−1^ of methylene blue (MB) in phosphate buffer (PB) was adjusted with HCl 1 M and NaOH 1 M to obtain solutions with controlled pH values ranging from 2 to 12. Each solution was contacted with the adsorbent material for 30 min under orbital shaking (250 rpm) at 25 °C. The solutions were then filtered through a 0.45 μm PTFE syringe filter. No adsorption of MB on the filter was detected. The UV–vis spectra taken in the range 400–900 nm and absorbance at 644 nm was recorded. The adsorption efficiency *AE* % was calculated according to Equation (1)

(1) $$AE\;\%=\;\frac{Abs_{0}-Abs}{Abs_{0}}\cdot 100,$$

where *Abs*_0_ and *Abs* are, respectively, the initial absorbance and the absorbance after treatment, measured at 664 nm.

#### 3.2.2. Effect of Adsorbent Dosage

The effect of adsorbent dosage on the adsorption efficiency was studied using 0.125, 0.25, 0.5, 1, and 2 g·L^−1^ amounts of adsorbent. The experiments were performed at pH 4 using the PB solution containing 50 mg L^−1^ of methylene blue. The same procedure described in Section 3.2.1 was followed.

#### 3.2.3. Effect of Adsorption Time

The effect of adsorption time on the adsorption efficiency was studied in a time interval from 5 to 60 min. The experiments were done at pH 4 using the PB solution containing 50 mg·L^−1^ of methylene blue. The same procedure described in Section 3.2.1 was followed. 

#### 3.2.4. Reuse of Adsorbent

The possibility of regenerating and thus reusing the sepia melanin-P(E-*alt*-MA) hybrid was tested by means of pH and ionic strength. After following the usual adsorption protocol (50 mg·L^−1^ MB, pH 4, 30 min, 25 °C, 250 rpm), the supernatant was discarded and replaced with 20 mL of NaOH 1 M, HCl 1 M, or NaCl 1 M. After 10 min of stirring at 250 rpm, the supernatant was discarded, the adsorbent washed with 20 mL of water three times (or until neutral pH) and the adsorption protocol with MB was repeated. 

## 4. Conclusions

By combining sepia melanin and poly(ethylene-*alt*-maleic anhydride), a hybrid material can be easily synthesized. Our hybrid displays promising adsorption properties, demonstrated against methylene blue, in a wide range of pH (2–12) and with fast kinetics (minutes instead of hours). Moreover, it can be recycled up to three times without a significant loss of efficiency. All these characteristics make our hybrid potentially useful for water purification purposes. 

## Figures and Tables

**Table 1:** Elemental percentages and ratios obtained by EDX.

P(E-*alt*-MA)		Sepia Melanin			Hybrid		
C (%)	O (%)	C (%)	O (%)	N (%)	C (%)	O (%)	N (%)
58 ± 0.73	41 ± 0.99	53 ± 1.3	35 ± 1.4	11 ± 2.5	58 ± 3.0	35 ± 3.9	7.2 ± 2.7
C/O 1.4		C/O 1.5			C/O 1.7		
		C/N 5			C/N 8		

**Table 2:** Residual mass values obtained from TGA.

Sample	Residual Mass (%)
P(E-*alt*-MA)	10.03
Sepia melanin	44.46
Hybrid	37.40

## Supplementary Materials

The following are available online at http://www.mdpi.com/2079-4991/8/2/54/s1. Figure S1: SEM image of a sepia melanin granule showing the spherical particles it is made of, Figure S2: FTIR spectra of sepia melanin, P(E-*alt*-MA) and of the sepia melanin-P(E-*alt*-MA) hybrid, Figure S3: Evaluation of the adsorption efficiency of sepia melanin for methylene blue as a function of pH. Conditions: 1 g L^−1^ of adsorbent, 50 mg L^−1^ of methylene blue, 30 min, 250 rpm, 25 °C.
