# Supplementary material for: Preparation of a Sepia Melanin and Poly(ethylene-alt-maleic Anhydride) Hybrid Material as an Adsorbent for Water Purification
Source: Nanomaterials (Basel). 2018 Jan 23;8(2):54. doi: 10.3390/nano8020054 (PMC5853687; doi:10.3390/nano8020054)
Supplement: Supplementary file 1 [file nanomaterials-08-00054-s001.pdf]

SUPPORTING INFORMATION

Preparation of a sepia melanin and poly(ethylene-*alt*-maleic anhydride) hybrid material as an adsorbent for water purification

Guido Panzarasa [1,*], Alina Osypova [2], Giovanni Consolati [3], Fiorenza Quasso [3], Guido Soliveri [4], Javier Ribera Regal [5] and Francis W. M. R. Schwarze [5]

1 Department of Polymer Engineering and Science, Montanuniversität, Otto-Glöckel Straβe 2, 8700 Leoben, Austria; gp4779@gmail.com
2 Sorbonne Universités, UPMC Univ Paris 06, F-75005, Paris, France; alina.osypova@gmail.com
3 Department of Aerospace Science and Technology, Politecnico di Milano, via La Masa 34, 20156 Milano, Italy; giovanni.consolati@polimi.it; fiorenza.quasso@polimi.it
4 Department of Chemistry, Université de Montréal, C.P. 6128 Succ. Centre-ville, Montréal, Quebec, H3C 3J7 Canada; guido.soliveri@gmail.com
5 Empa Materials Science and Technology, Laboratory for Applied Wood Materials, Lerchenfeldstrasse 5, 9014 St. Gallen, Switzerland; javier.ribera@empa.ch; francis.schwarze@empa.ch

* Correspondence: gp4779@gmail.com; Tel.: +41-079-679-1994

Academic Editors: Dr. Daniela Meroni and Prof. Silvia Ardizzone.

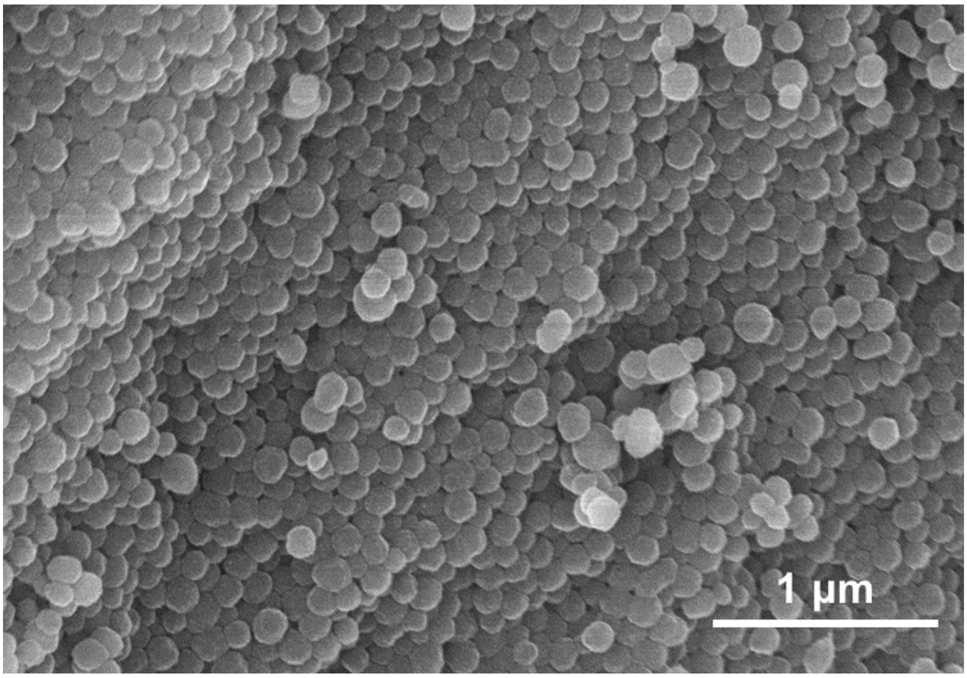

Figure S1. SEM image of a sepia melanin granule showing the spherical particles it is made of.

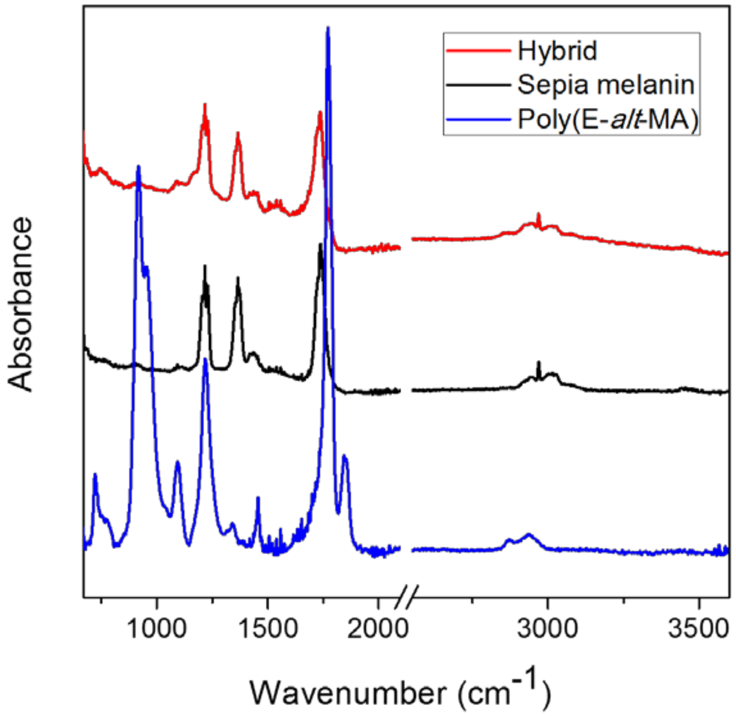

Figure S2. FTIR spectra of sepia melanin, P(E-*alt*-MA) and of the sepia melanin-P(E-*alt*-MA) hybrid.

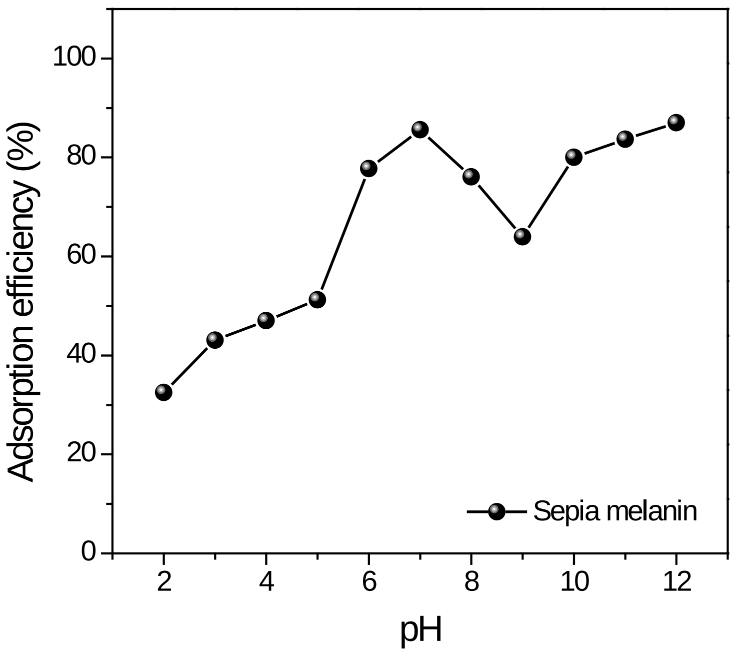

Figure S3. Evaluation of the adsorption efficiency of sepia melanin for methylene blue as a function of pH. Conditions: 1 g L^{-1} of adsorbent, 50 mg L^{-1} of methylene blue, 30 min, 250 rpm, 25°C.
